# Precise Au_x_Pt_1−x_ Alloy Nanoparticle Array of Tunable Composition for Catalytic Applications

**DOI:** 10.1038/srep20536

**Published:** 2016-02-09

**Authors:** Sarah Jahn, Sebastian J. Lechner, Helene Freichels, Martin Möller, Joachim P. Spatz

**Affiliations:** 1 Department of New Materials and Biosystems, Max Planck Institute for Intelligent Systems, Stuttgart, Germany; 2 Department of Biophysical Chemistry, University of Heidelberg, Heidelberg, Germany; 3 DWI - Leibniz Institute for Interactive Materials, Aachen, Germany

## Abstract

A 3-dimensional Block Copolymer Micellar nanoLithography (BCML) process was used to prepare Au_x_Pt_1−x_ alloy nanoparticles (NPs) monodisperse in size and composition, strongly anchored onto SiO_2_-particles (0.2 wt.% Au_x_Pt_1−x_/SiO_2_). The particles possess a face-centered cubic (fcc) crystal structure and their size could be varied from 3–12 nm. We demonstrate the uniformity of the Au/Pt composition by analyzing individual NPs by energy-dispersive X-ray spectroscopy. The strongly bound Au_x_Pt_1−x_ NPs catalyzed the oxidation of CO with high activity. Thermal ageing experiments in pure CO_2_ as well as in ambient atmosphere demonstrated stability of the size distribution for times as long as 22 h.

Noble metal nanoparticles (NPs) made from Au and Pt find application in many areas of surface chemistry as well as for biotechnological assays and methods^1^,^2^,^3^. Their specific properties, e.g., in catalytic reactions such as the electrochemical oxidation of methanol^4^ and the reduction of CO.^5^,^6^,^7^,^8^,^9^, depend on both intrinsic and external factors. The former include the NP’s phase behavior and structure as well as its composition and size distribution, the latter refer to the influences stemming from the substrate, dispersion, and the environmental conditions of the reaction^5^,^10^. Many applications in nanotechnology harness the well-known fact that NPs made from a combination of two different noble metals have favorable properties concerning their thermal stability, catalytic performance as well as optical and magnetic properties compared to NPs made from pure metals^6^,^11^,^12^. As an example, Ethirajan *et al.*^13^ and Wiedwald *et al.*^14^ were able to synthesize FePt and CoPt alloy NPs with superior magnetic properties via Block-Copolymer Micellar nanoLithography (BCML). The structure of such mixed NPs depends on the synthesis method and the kinetics of the reaction. Thus, the Au-Pt phase diagram exhibits a miscibility gap in the bulk state^15^, however at the nanoscale, Au and Pt atoms can form stable bimetallic NPs in a core shell or an alloy structure^16^.

Concerning the catalytic activities and properties of Pt, Au, and AuPt on different substrates and under various conditions, Haruta *et al.* have identified Au as a very active catalyst during low temperature CO oxidation^5^,^7^. These findings have also been confirmed in studies by other authors, which found the catalytic activity of Au NPs (2–4 nm in diameter) to be much higher than that of Pt NPs^6^,^8^,^9^. However, the low thermal stability of small Au NPs (approximately <5 nm) as well as its rapid decrease in catalytic activity with increasing NP size have, thus far, restricted the use of Au in industrial applications. In contrast, Pt NPs exhibit constant catalytic activity over a wide range of particle sizes. Here, Au_x_Pt_1−x_ NPs can have an advantage by combining high activities in catalytic reactions with improved stability^6^,^12^. AuPt nanoalloys have greater catalytic activity and thermal stability than pure Au or Pt^6^. In addition, Yamamoto *et al.*^17^ have reported an improved performance of AuPt compared to pure Pt during CO oxidation^6^. Still, there is much room for improving the catalytic behavior of noble metal NPs concerning their performance as low temperature catalysts, their sinter behavior, and their stability. Just as important for their industrial application is also an improved understanding of the role of nonmetallic substrates^10^.

In this work, Au_x_Pt_1−x_ NPs were synthesized and immobilized on 2D and 3D substrates, more specifically Y:ZrO_2_ wafers and mesoporous silica powder, using BCML. The BCML technique enables the synthesis of NPs with tunable and reproducible composition as well as high monodispersity. In addition, due to the self-assembling character of the loaded micelles, Au_x_Pt_1−x_ NPs can be distributed uniformly and in a predictable hexagonal arrangement on the substrate. Multiple properties of the substrate-bound NPs – including their size and dispersity as well as the crystal structure and composition of alloy NPs –, were analyzed by high-resolution transmission electron microscopy (HRTEM), high-angle annular dark-field imaging (HAADF), energy-dispersive X-ray spectroscopy (EDX), and inductively coupled plasma atomic emission spectroscopy (ICP-AES). CO oxidation studies of monodisperse Au, Pt, and Au_x_Pt_1−x_ alloy NPs were conducted to investigate correlations between the properties of the catalyst and its catalytic activity. Catalytic activity measurements of the NPs were performed by way of a cyclic temperature-programmed CO oxidation reaction.

## Results

### BCML of bimetallic alloy NPs

Au_x_Pt_1−x_ NPs were synthesized using a previously described block copolymer micellar nanolithography technique (BCML)^18^,^19^,^20^,^21^,^22^. The size of the NPs and the distance between individual NPs was tailored through adjusting the choice of the block copolymer, the concentration of the micellar solution, and the metal loading. The amount of metal salt in the micelles is a function of the loading parameter L:


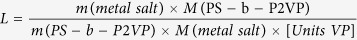


where m represents the mass, M the molar mass and [Units VP] the amount of vinylpyridine monomer. Block copolymers can form self-assembled monomicellar layers. Flat substrates can be spin- or dip-coated by a monomicellar film whereby the noble metal salt is concentrated within the core of the micelles. By a subsequent plasma treatment monodisperse noble metal NPs deposite in a quasi-hexagonal pattern at the interface of the substrate^18^,^19^,^20^. To generate bimetallic alloy noble metal NPs, the micelles are loaded by two different metal salts, a second loading step is applied after the first loading step (M_A_). The metal ions of the second metal salt (M_B_) also diffuse into the hydrophilic core of the micelle. M_A_M_B_ NPs are formed during the following plasma treatment^23^.

**Figure 1:**
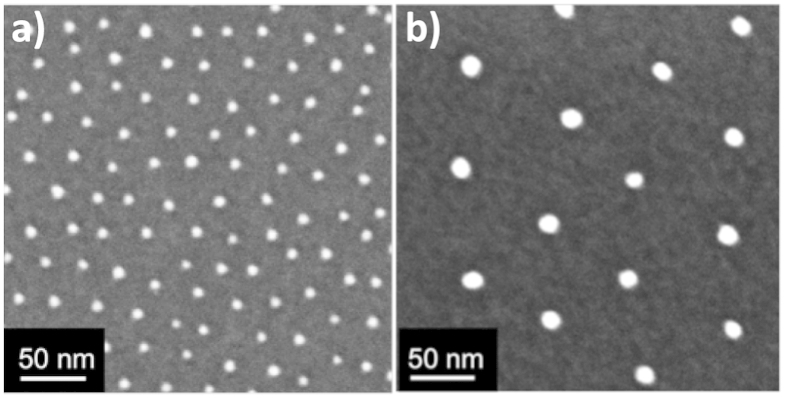
SEM recorded with back-scattered electrons of alloy NPs. The images depict Au_0.5_Pt_0.5_ alloy NPs with different particle sizes [(**a**) 6 nm, (**b**) 10 nm] and interparticle distance [(**a**) 20 nm, (**b**) 70 nm] as produced using two different block copolymers [(**a**) PS(227)-b-P2VP(99) and (**b**) PS(1056)-b-P2VP(495)]. The concentration of the micellar solutions was identical: c = 3 mg/ml, loading L = 0.4.

Employing this procedure, we were able to synthesize alloy NPs with diameters between 3–12 nm and desired noble metal ratios Au_x_Pt_1−x_. The following noble metal ratios were used for the catalytic measurements: Au_0.1_Pt_0.9_, Au_0.3_Pt_0.7_ and Au_0.5_Pt_0.5_. Pure Au NPs and Pt NPs were also compared. The optimization of the diameter, particle size, and interparticle distance of Au_x_Pt_1−x_ alloy NPs based on the utilization of different block copolymers is shown in Fig. 1 using the example of Au_0.5_Pt_0.5_NPs.

For catalytic applications a sufficiently large number of noble metal NPs must be well-dispersed on a carrier with a high surface to volume ratio in order to enable sufficient chemical conversions. This can typically be realized as a washcoat. Washcoats are ceramic powders, comprised typically of SiO_2_, TiO_2_, Al_2_O_3_, or mixtures of these materials, which cover the walls of the honeycomb structures of catalytic converters^24^,^25^. Here, mesoporous silica powder is used as a substrate for the immobilization of the Au_x_Pt_1−x_ NPs. The total surface area was calculated from an adsorption-desorption isothermal curve obtained from H adsorption experiments. The silica powder had a total surface area of a_BET_ = 141.1 m^2^/g.

**Figure 2:**
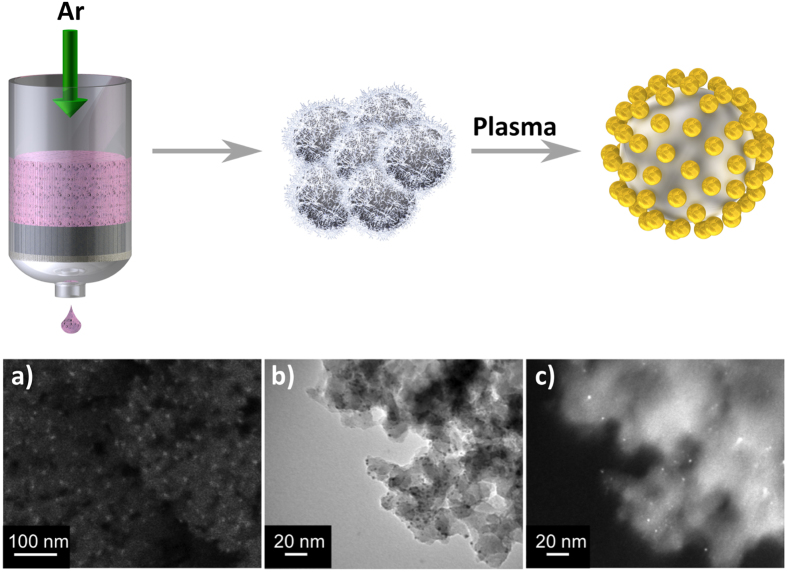
Scheme of the 3D coating process for ceramic powder particles. The micellar solution loaded with metal salt is mixed with the silica powder and pressed through the powder particles by Argon flux. The micelles form a monolayer covering the entire surface area of the powder particles. After plasma treatment, which burns the polymeric parts and reduces the metal salt to M^0^, NPs are immobilized on the substrate: (**a**) SEM (ESB), CTEM (**b**) BF and (**c**) DF of Au_0.5_Pt_0.5_ NPs immobilized on silica powder (polymer: PS(227)-b-P2VP(99)).

Gadomska *et al.*^26^ established a method for creating well-ordered gold NPs on spheres called BCML. Employing their BCML method, they covered 75 μm glass beads with a monolayer of quasi hexagonally-ordered micelles. Likewise, it is possible to coat the surface of mesoporous silica powder with Au_x_Pt_1−x_ alloy NPs. The SEM and CTEM characterization of the coated powder showed excellent dispersion of the Au_x_Pt_1−x_ alloy NPs on the surface of the ceramic powder particles (Fig. 2).

The following analysis and measurement of the catalytic activity in a CO oxidation reaction was performed on Au_x_Pt_1−x_ alloy NPs (approx. 6 nm) synthesized via BCML using the PS(227)-b-P2VP(99) block copolymer. For the size distribution of the Au_x_Pt_1−x_ NPs used for catalytic measurements and characterization see Supplementary Fig. S1 and S2 online.

**Figure 3:**
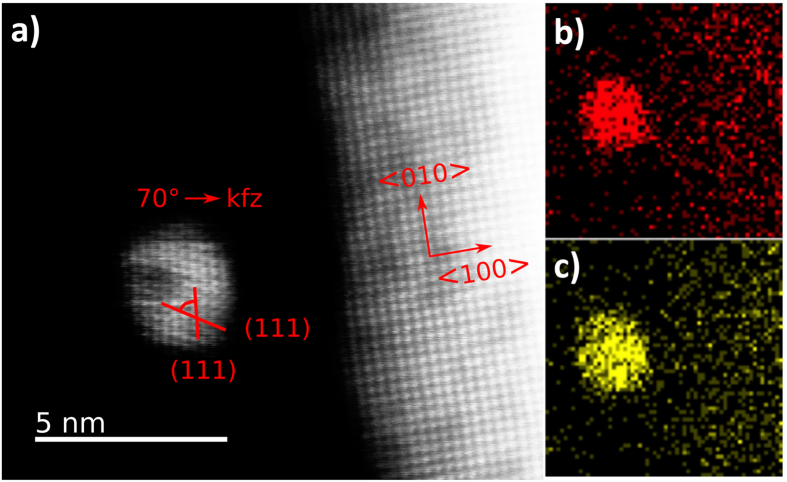
Au_0.5_Pt_0.5_ NP on Y:ZrO_2_ (100): (**a**) HAADF Image (200 kV) and EDX mapping of (**b**) Au (L) and (**c**) Pt (L). Au and Pt atoms are randomly distributed in the NP.

The Au_x_Pt_1−x_ NPs synthesized using BCML were analyzed by high-resolution transmission electron microscopy (HRTEM), high-angle annular dark-field imaging (HAADF), energy-dispersive X-ray spectroscopy (EDX), and inductively coupled plasma atomic emission spectroscopy (ICP-AES). Our measurements revealed that Au and Pt atoms are randomly distributed inside the NPs (see Fig. 3). Concerning their atomic lattice parameters, the NPs are arranged along the <110> direction and show two (111) lattice planes. The angle between the two (111) lattice planes is 70°, which indicates a face-centered cubic (fcc) crystal structure. The results are in agreement with recent work by Petkov *et al.*^27^ who observed a random alloy structure in Au_x_Pt_1−x_ NPs (<10 nm) synthesized by a two-phase method using aqueous solutions of the metal salts.

EDX measurements of single NPs and ICP-OES measurements of the coated silica powder were performed to quantify the atomic ratio of Au to Pt. The NPs designated for catalytic CO oxidation measurements (i.e., Au_0.1_Pt_0.9_, Au_0.3_Pt_0.7_, and Au_0.5_Pt_0.5_NPs) were analyzed. Results are listed in Table 1. The alloy composition in the left column states the originally used Pt and Au amounts. Most importantly, the measurement of the atomic ratio within the overall sample, shown in the column on the right, proves that the designated alloy composition is achieved not only in some but in all NPs. We believe that the precision of this production method with regard to obtaining a particular metal ratio in every single NP is outstanding and cannot be reproduced by any other method for preparing alloy nanoparticles.

**Figure 4:**
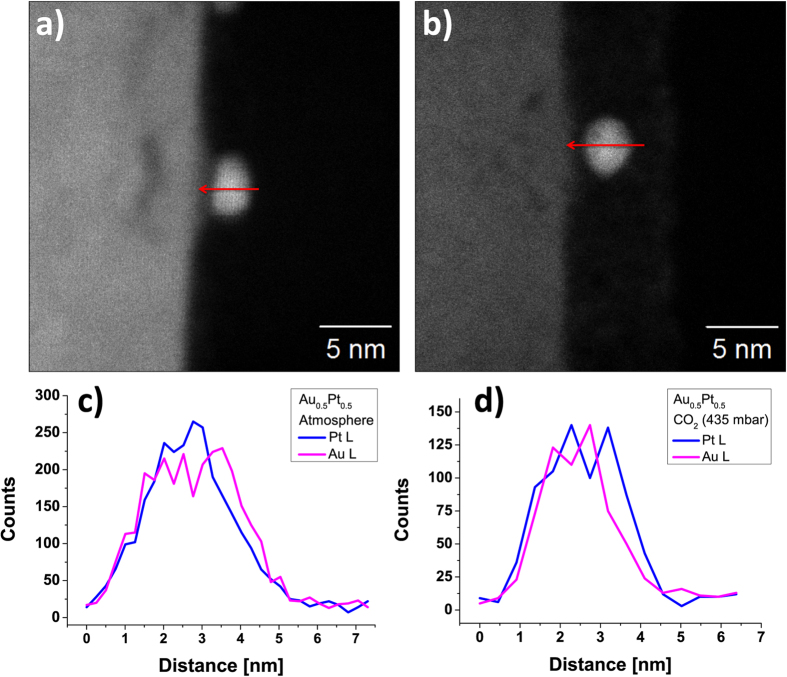
HAADF image of Au_0.5_Pt_0.5_ NPs on Y:ZrO_2_ (100) after annealing for 22 h at 400 °C in (**a**) atmosphere and (**b**) CO_2_ (both 435 mbar). The samples shown in the EDX linescans (red arrow indicates the scanning direction) were annealed under (**c**) atmosphere or (**d**) CO_2_. They do not show any phase segregation of Au and Pt atoms after the aging process.

Regarding the thermal stability of the Au_x_Pt_1−x_ alloy NPs EDX measurements were performed to verify that also no phase segregation of Au and Pt takes place. For this, Au_0.5_Pt_0.5_NPs were immobilized on Y:ZrO_2_ (100) planar substrates and aged under CO_2_ (435 mbar) and atmospheric conditions at 400 °C for 22 hours (Fig. 4). The EDX results reveal that phase segregation does not occur during the aging process. Furthermore, no changes in the size distribution of all used alloy (Au_x_Pt_1−x_) and pure noble metal (Au, Pt) NPs were observed.

ICP-AES analysis not only reveals the ratio of the compounds but also provides information about the quantity of noble metal immobilized on the surface of the silica powder. Au_x_Pt_1−x_ samples of each ratio were prepared using an identical procedure. Our measurements showed that in all cases the amount of noble metal dispersed on the surface of the silica powder constituted about 0.2 wt% of the sample.

Experiments pertaining to the catalytic activity in the CO oxidation reaction were performed in a fully automated set-up, thus allowing the measurement of transient kinetics. Helium was used as the balance gas. The reactor was operated with a theoretical oxygen excess of 100% for the reaction 
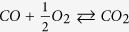
.

**Figure 5:**
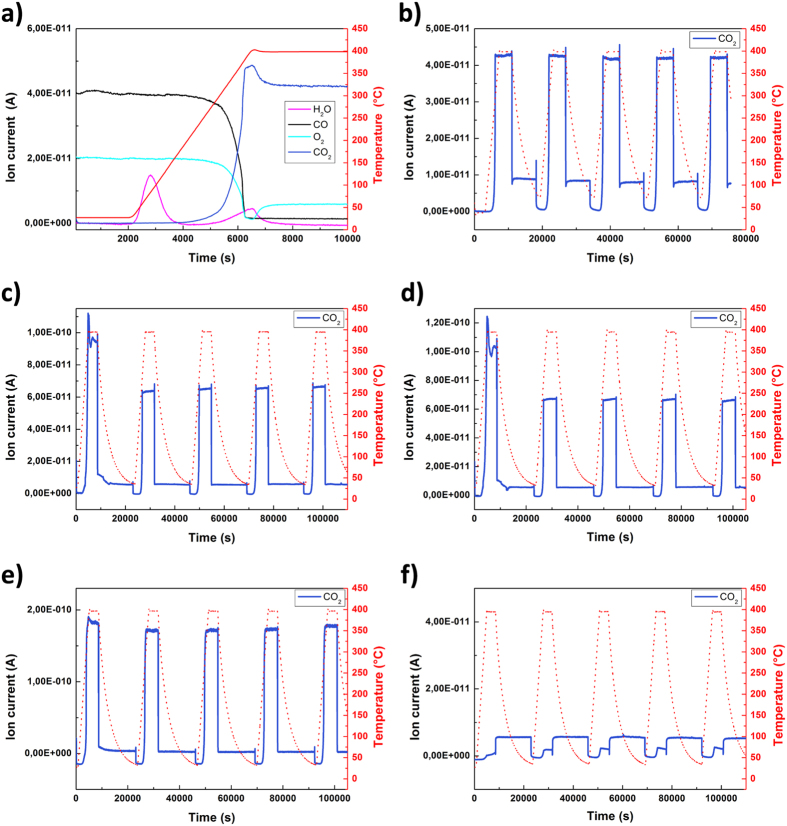
Temperature-controlled CO oxidation between 25 and 400 °C. The graphs show the first temperature rise with an overview of all measured signals for (**a**) 0.2%Au_0.5_Pt_0.5_/SiO_2_ and the course of the CO_2_ signal of 5 measured cycles for (**b**) 0.2%Au_0.5_Pt_0.5_/SiO_2_, (**c**) 0.17%Au_0.3_Pt_0.7_/SiO_2_, (**d**) 0.18%Au_0.1_Pt_0.9_/SiO_2_, (**e**) 0.15%Pt/SiO_2_, and (**f**) 0.23%Au/SiO_2_.

It is well known that Pt surfaces show a strong tendency for CO poisoning^28^; therefore, CO-rich conditions were chosen in order to evaluate the possible contribution of the gold content in CO oxidation. Temperature-programmed oxidation was carried out cyclically with a total volume flow rate of 20 NmL/min and a heating rate of 5 °C/min from 25–400 °C. Ion currents of the molecular ions of CO_2_, CO, O_2_, and H_2_O were evaluated. The ion current of CO was corrected by the theoretical ratio of the produced CO_2_ (which equals 11,4%), because CO is a fragment of CO_2_. The results of the CO oxidation measurements are displayed in Fig. 5. The result of the first heating cycle of the 0.2%Au_0.5_Pt_0.5_/SiO_2_ NPs is of particular importance (see Fig. 5a): during this first stage of heating (up to nearly 300 °C), the amount of CO decreased strongly. Simultaneously, the O_2_ signal decreased to zero before slightly increasing to a constant value at higher temperatures. The CO_2_ signal – the product of the oxidation reaction – reached a peak before arriving to a steady state regime. The turnover rate of CO oxidation reaches a limiting value determined by the rate of adsorption-desorption of the involved molecules^29^,^30^. Furthermore, two clearly separated H_2_O peaks are visible, one below 100 °C and one at approximately 400 °C. The first peak indicates a significant amount of weakly bound, physisorbed water, whereas the second peak, which is also lower in intensity, suggests strongly bound water. The water peaks only appear during the initial heating of the samples. For the sake of simplicity, only the CO_2_ signal and the temperature profile of 5 cycles are plotted for the other Au_x_Pt_1−x_ NPs-based catalysts (Fig. 5b–f).

**Figure 6:**
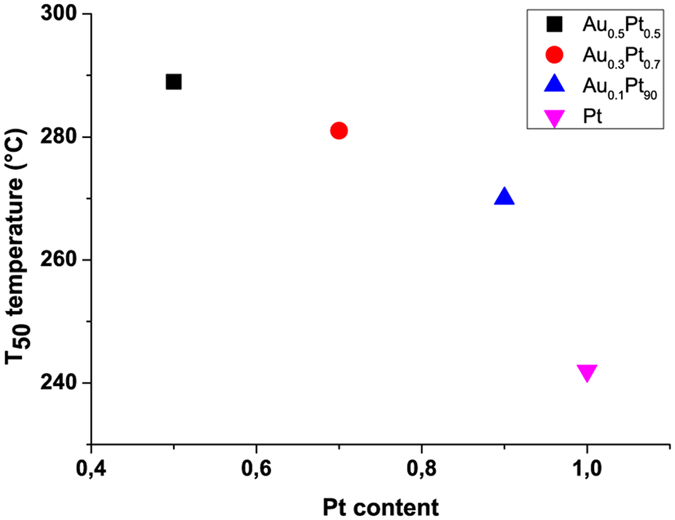
T_50_ temperature related to the Pt content of the Au_x_Pt_1−x_ NPs.

The catalytic behavior of all Au_x_Pt_1−x_/SiO_2_ NP samples was similar with the exception of the 0.23%Au/SiO_2_ sample. The T_50_ temperature, at which 50% of the CO streamed in is turned over, is a direct indicator of the catalytic activity. Table 2 gives an overview of the catalytic activity of the five measured cycles. During the first cycle, activation of the catalyst takes place. This activation is demonstrated by the observed removal of stored molecules and water from the Pt sample (see Fig. 5a). Therefore, the T_50_ temperature of the first cycle is much higher than in later cycles, but reaches a constant value of catalytic activity with the second cycle. Additionally, the 0.17%Au_0.3_Pt_0.7_ and 0.18%Au_0.1_Pt_0.9_ samples show a higher CO_2_ discharge during the first cycle compared to the other samples. This higher amount of CO_2_ discharge could be due to the reaction of stored hydrocarbons on the samples, which also contribute to the signal. The stable T_50_ values of the subsequent cycles indicate no ageing of the samples for temperature cycles up to 400 °C. The T_50_ temperature of the fifth cycle for each sample is used to compare the catalytic performance in CO oxidation (Table 2). The pure 0.15%Pt/SiO2 sample showed the highest catalytic activity of all the tested samples with a T_50_ temperature of 242 °C after activation. In contrast, the 0.23%Au/SiO2 sample showed no catalytic activity under these experimental and evaluation conditions. We do not measure any advantage of Au on Pt-based NPs with respect to improving the catalytic performance during CO oxidation. The correlation between the T_50_ temperature and NP composition is most evident: T_50_ increases with increasing amounts of Au and decreasing amounts of Pt in the NPs (Fig. 6). In particular, a comparison of the 0.15%Pt and 0.18%Au_0.1_Pt_0.9_ samples clearly shows that, although the amount of Pt is similar in both NPs, the T_50_ temperature is decidedly different. This finding suggests that Au atoms compete with Pt atoms at the NP surface, which, in turn, reduces the overall catalytic activity of the NPs with increasing amount of Au. In general, the catalytic activity of the Au_x_Pt_1−x_ NPs is in agreement with the published activity of pure Pt catalysts^31^,^32^.

In other words, the observed T_50_ for CO oxidation is closer to that of pure Pt catalysts than to the T_50_ reported for pure Au/support catalysts in ambient temperature ranges^32^.

## Discussion

BCML proved to be a powerful tool to synthesize monodisperse fcc alloy crystal structure NPs of desired composition, size, and interparticle distance with excellent precision. Most importantly, the ratio between Pt and Au that was measured in individual NPs was very consistent between the sampled NPs. It was possible to produce 0.2% Au_x_Pt_1−x_/SiO_2_ model catalysts by using a 3D BCML coating process. The catalytic activity of these Au_x_Pt_1−x_/SiO_2_ systems during CO oxidation was tested by temperature-controlled oxidation. The level of oxidation activity was determined on the basis of the T_50_ temperature and the systems ranked according to their activity. The activity of each catalyst as prepared by BCML did not show any ageing effects for temperatures up to 400 °C. The T_50_ temperature revealed further that catalytic activity correlates with the amount of Pt in the NP. We assume that the observed changes in oxidation activity are due to a higher surface activity of Au atoms on the surface of alloy NPs as Au atoms compete with Pt atoms for surface occupation. However, no core shell structure or other phase segregation structures could be discovered in our alloy NPs. The ability to selectively compose individual metal alloy NPs with high precision opens up new possibilities to study the catalytic performance of alloy noble metal catalysts. It greatly improves the understanding of the role that individual NP components play. Additionally, the role of the support could be the focus of future catalytic experiments, in which the interaction between the support and the noble metal NPs, known as the “Strong Metal-Support Interaction” (SMSI), and the reactivity of the perimeter interface could be further examined. Specifically, supports like TiO_2_ or CeO_2_ are known to enhance the catalytic activity of noble metal NPs^5^,^10^,^33^,^34^. With this knowledge further catalytic applications can be considered.

## Methods

### Synthesis and deposition of NPs in 2D and 3D

The block copolymer PS(227)-b-P2VP(99) was dissolved in toluene at a concentration of 3 mg/ml and stirred for 24 h. Metal salt loading of the micelles in the solution was performed at L = 0.4. The loading ratios of both metal salts was adjusted individually to produce alloy nanoparticles of different metal compositions. For instance, to compose Au_0.5_Pt_0.5_NPs, for which equal parts of gold and platinum were used, the loading ratios of Gold(III)chloridetrihydrate (HAuCl4 • 3H_2_O) and Hexachloroplatinic(IV) acid hexahydrate (H_2_PtCl6 • 6H_2_0) were both set to L = 0.2, thus achieving a total loading ratio of L = 0.4. Due to the fact that Au ions stabilize micelles, Au salt was added to the solution and dissolved before adding the Pt salt. The loaded micelles were immobilized on the surface of a Y:ZrO_2_ (100) wafer by spin coating the solution at 6000 rpm. In order to remove the polymer and reduce the metal salt, the coated wafer was then treated with Hydrogen (10%)-Argon (90%) plasma (45 min, 350 W, 0.4 mbar).

To immobilize the Au_x_Pt_1−x_ NPs on the silica powder (mean density of 300–400 g/l), 3 g of silica powder was mixed with 15 ml of the loaded micellar solution and sonicated for 30 seconds. The wet powder was then put into a glass column sealed with a PFTE frit (pore size 30 μm) and pressed through the powder using an Argon flux. After drying, the powder was treated with pure H-plasma (45 min, 300 W, 0.4 mbar).

### Annealing

The Au_0.5_Pt_0.5_ NPs immobilized on Y:ZrO_2_ (100) wafers were annealed in air or under CO_2_ atmosphere for 22 h at 400 °C (heating rate of 5 K/min). The samples in the CO_2_ atmosphere were evacuated in a glass tube filled with CO_2_ (435 mbar/RT).

### Characterization

The arrangement and order of the NPs on the substrates in 2D and 3D was analyzed by SEM (Zeiss Ultra 55, 2 and 5 kV). The in-lens and ESB detector were used for imaging. The size distribution of the Au_x_Pt_1−x_ NPs was measured using TEM (CM 200, Philips, 200 kV). For this, the NPs were immobilized on TEM Cu grids with a SiO_2_ membrane (mesh 300, 20 nm, Plano GmbH). To analyze the NPs immobilized on silica powder, a small amount of the sample was mixed with Ethanol and 5 μl was deposited onto TEM grids (Cu mesh 400) with a carbon film substrate. The TEM measurements to analyze the size distribution as well as the visual analysis of the powder structure were performed on a Philips 200 kV Transmission Microscope in BF, DF, and diffraction mode. EDX/HR-EDX measurements and HAADF imaging were performed on the SESAM (Sub-Electron-volt-Sub-Angstrom TEM, Zeiss, 200 kV) and ARM (JEOL-ARM200CF, 200 kV) microscopes. The EDX measurements of the NPs were performed on the same samples used for size distribution measurements. The crystal structure of the Au_0.5_Pt_0.5_ NPs was measured by preparing cross-cut samples of single NPs on a Y:ZrO_2_ (100) wafer employing a PIPS (precision ion polishing system). The composition of the NPs was also examined by EDX mapping, line scans, and spectra over single NPs. The TEM measurements were performed at the Stuttgart Center for Electron Microscopy (StEM).

### Catalytic Measurements

The catalytic measurements were performed at RUBOKAT GmbH, Bochum. The CO oxidation reaction was performed in a fully automated reactor capable of measuring transient kinetics (BELCAT B; BEL corp. Japan). Specifically, ion currents of CO_2_, CO, O_2_, and H_2_O molecular ions were measured. The reactor is attached to a gas mixing unit (BEL (J)) that uses helium as a diluent gas. The gas mixing unit was used to adjust the CO flow so that 1% CO gas was mixed with 1% O_2_ gas. The reactor was operated with a theoretical oxygen excess of 100% for the reaction 
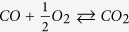
.

Temperature-controlled oxidation was carried out cyclically, with a total volume flow rate of 20 Nml/min and a heating rate of 5 K/min from 25–400 °C. The initial weight was about 100 mg per catalytic measurement. The detector consisted of an online quadrupole mass spectrometer (Pfeiffer, GAM 400). The ion current of CO was corrected with the theoretical ratio of 11,4% of the produced CO_2_, because CO is a fragment of CO_2_.

## Additional Information

**How to cite this article**: Jahn, S. *et al.* Precise Au_x_Pt_1−x_ Alloy Nanoparticle Array of Tunable Composition for Catalytic Applications. *Sci. Rep.* **6**, 20536; doi: 10.1038/srep20536 (2016).

## Supplementary Material

Supplementary Information

## Figures and Tables

**Table 1:** EDX and ICP-AES characterization of Au_x_Pt_1−x_ NP.

Au_x_Pt_1-x_	EDX [At.%]	ICP [At.%]
Au_0.5_Pt_0.5_	Au_0.48_Pt_0.52_ ± 0.06	Au_0.51_Pt_0.49_
Au_0.3_Pt_0.7_	Au_0.27_Pt_0.73_ ± 0.09	Au_0.3_Pt_0.7_
Au_0.1_Pt_0.9_	Au_0.13_Pt_0.87_ ± 0.05	Au_0.16_Pt_0.84_

**Table 2:** T_50_ temperatures of the first 5 cycles of all tested Au_x_Pt_1-x_/SiO_2_ samples.

Cycle	0.2%Au_0.5_Pt_0.5_ T_50_ [°C]	0.17%Au_0.3_Pt_0.7_ T_50_ [°C]	0.18%Au_0.1_Pt_0.9_ T_50_ [°C]	0.15%Pt T_50_ [°C]	0.23%Au T_50_ [°C]
1	356	337	321	319	<400
2	289	283	273,5	243	<400
3	290	282	271	241	<400
4	292	281,5	270,5	241	<400
5	289	281	270	242	<400
