# Supplementary material for: Precise AuxPt1−x Alloy Nanoparticle Array of Tunable Composition for Catalytic Applications
Source: Sci Rep. 2016 Feb 9;6:20536. doi: 10.1038/srep20536 (PMC4746634; doi:10.1038/srep20536)
Supplement: Supplementary Information [file srep20536-s1.pdf]

Supplementary Information

Precise Au_xPt_{1-x} Alloy Nanoparticle Array of Tunable Composition for Catalytic Applications

Sarah Jahn†,‡, *Sebastian J. Lechner*†,‡,§, Helene Freichels,§ *Martin Möller*§, and

Joachim P. Spatz†,‡*

†Department of New Materials and Biosystems, Max Planck Institute for Intelligent Systems, Stuttgart, Germany

‡ Department of Biophysical Chemistry, University of Heidelberg, Heidelberg, Germany

§DWI - Leibniz Institute for Interactive Materials, Aachen, Germany

Results

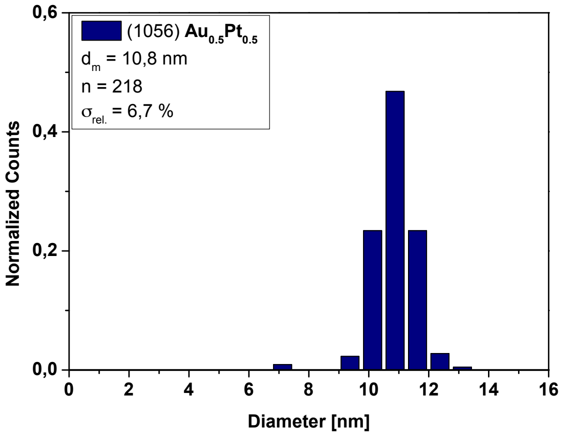

Figure S1. Size distribution of $Au_{0.5}Pt_{0.5}$ NP (Polymer: PS(1056)-b-2VP(495)).

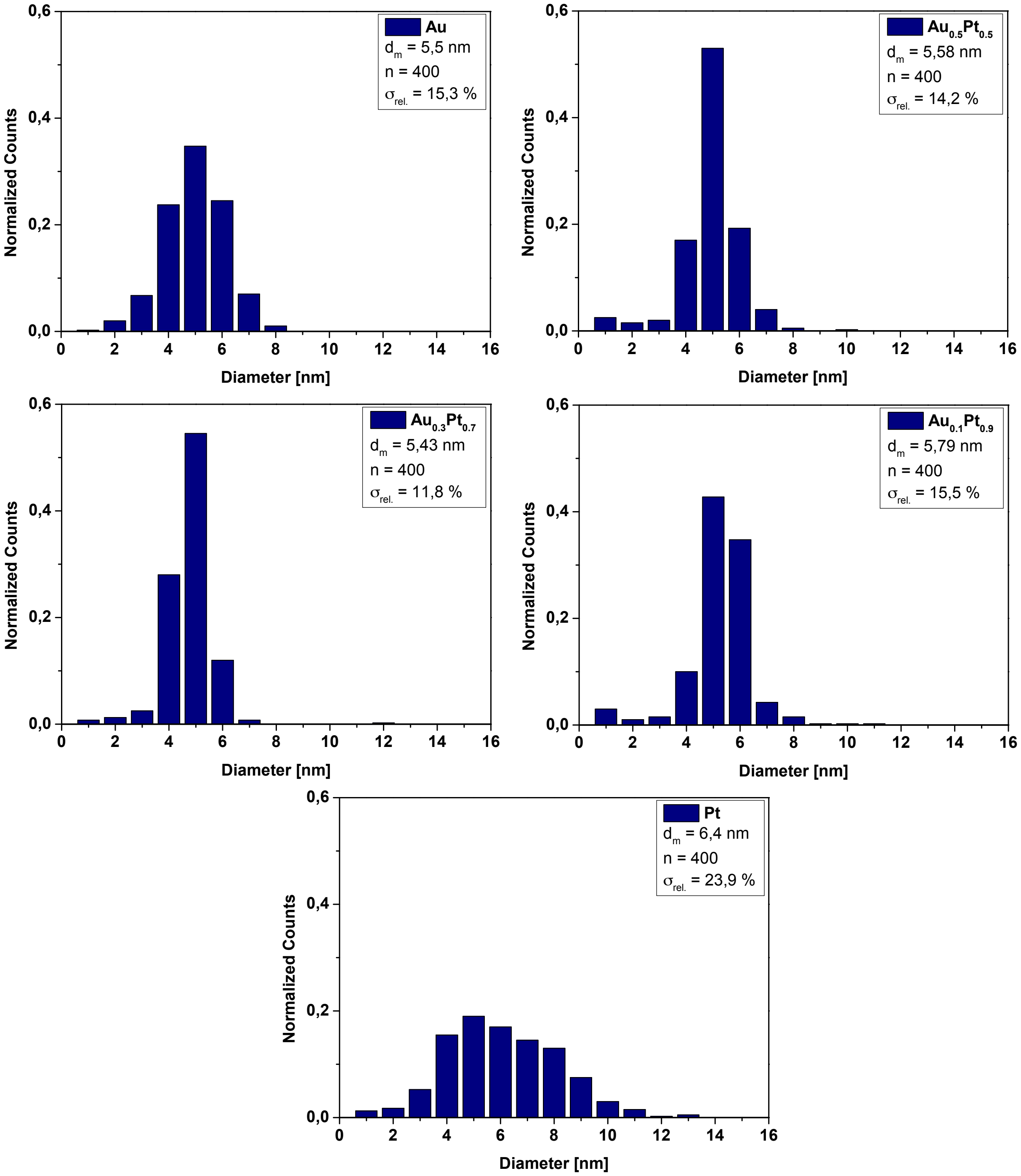

Figure S2. Size distribution of the Au_xPt_{1-x} NP used for catalytic measurements (Polymer: PS(227)-b-2VP(99)).
